# Clinical and biomechanical factors associated with falls and rheumatoid arthritis: baseline cohort with longitudinal nested case–control study

**DOI:** 10.1093/rheumatology/keab388

**Published:** 2021-04-27

**Authors:** Toby O Smith, Celia Clarke, Jacob Wells, Jack R Dainty, Laura Watts, Max Yates, Valerie M Pomeroy, Emma Stanmore, Terence W O’Neill, Alexander J Macgregor

**Affiliations:** 1 Faculty of Medicine and Health Sciences, University of East Anglia, Norwich; 2 Nuffield Department of Rheumatology, Orthopaedics and Musculoskeletal Sciences, University of Oxford, Botnar Research Centre, Oxford; 3 Rheumatology Department, Norfolk and Norwich University Hospital, Norwich; 4 Department of Clinical Neurosciences, NIHR Brain Injury MedTech Co-Operative, University of Cambridge, Cambridge; 5 Division of Nursing, Midwifery & Social Work, School of Health Sciences, Faculty of Biology, Medicine and Health, University of Manchester; 6 Centre for Epidemiology versus Arthritis, University of Manchester, NIHR Manchester Biomedical Research Centre, Manchester University NHS Foundation Trust, Manchester Academic Health Science Centre, Manchester, UK

**Keywords:** RA, falls, gait analysis, muscle strength, postural control

## Abstract

**Objective:**

To identify the clinical and biomechanical characteristics associated with falls in people with RA.

**Methods:**

A total of 436 people ≥60 years of age with RA completed a 1 year prospective survey of falls in the UK. At baseline, questionnaires recorded data including personal and medical history, pain and fatigue scores, health-related quality of life (HRQoL), physical activity and medication history. The occurrence of falls wasmonitored prospectively over 12 months by monthly self-reporting. A nested sample of 30 fallers (defined as the report of one or more falls in 12 months) and 30 non-fallers was evaluated to assess joint range of motion (ROM), muscle strength and gait parameters. Multivariate regression analyses were undertaken to determine variables associated with falling.

**Results:**

Compared with non-fallers (*n* = 236), fallers (*n* = 200) were older (*P* = 0.05), less likely to be married (*P* = 0.03), had higher pain scores (*P* < 0.01), experienced more frequent dizziness (*P* < 0.01), were frequently taking psychotropic medications (*P* = 0.02) and reported lower HRQoL (*P* = 0.02*).* Among those who underwent gait laboratory assessments, compared with non-fallers, fallers showed a greater anteroposterior (AP; *P* = 0.03) and medial-lateral (ML) sway range (*P* = 0.02) and reduced isokinetic peak torque and isometric strength at 60° knee flexion (*P* = 0.03). Fallers also showed shorter stride length (*P* = 0.04), shorter double support time (*P* = 0.04) and reduced percentage time in swing phase (*P* = 0.02) and in knee range of motion through the gait cycle (*P* < 0.01).

**Conclusion:**

People with RA have distinct clinical and biomechanical characteristics that place them at increased risk of falling. Assessment for these factors may be important to offer more targeted rehabilitation interventions.

**Rheumatology key messages**

- Clinical and biomechanical factors are useful to identify people with RA at risk of falls.
- Age, disease severity and psychotropic medications are key factors associated with the risk of falls.
- Exercise programmes targeting gait and strength deficits rather than overall physical activity may be indicated.

## Introduction

Falls are a major health and social care challenge worldwide [1]. The aetiology is multifactorial, with an interaction between intrinsic, behavioural and environmental factors [2]. In addition to the associated injury risk and loss of confidence and independence, falls and subsequent fractures are a significant cause of illness and death in older people [1–3].

RA affects ∼1% of the UK adult population [4]. It results in significant morbidity and increased healthcare costs [5, 6]. The prevalence of falls for people with RA has been reported to range from 10 to 43% [2], with Stanmore *et al*. [7] reporting an incidence rate of 1313 per 1000 person-years. Older people with RA may be at particular risk of falls and fracture due to disease-related factors such as pain, joint deformity, decreased muscle strength and osteoporosis associated with long-term steroid use [8].

A number of clinical factors have been associated with falls in older people. These include increased BMI [9], increasing number of comorbidities and polypharmacy [10], a history of falls, pain and fatigue [11, 12]. However, there remains uncertainty as to which specific clinical factors are associated with falls in people with RA. Furthermore, it remains unclear if specific biomechanical differences exist between individuals with RA who fall compared with those who do not experience falls. Previous biomechanical assessments of people with RA have focussed on foot and ankle function [13–16] and gait comparisons with healthy controls [17]. However, no studies have evaluated more global, kinematic features that may be associated with falls, such as gait speed [18]. Given that such kinematic measures differ in people with RA compared with non-RA cohorts [19], it is important to see if these could be associated with the risk of falls in this population. If shown to be associated, interventions to target specific deficits may be warranted.

Given this uncertainty in the RA population, this study aimed to characterize the clinical and biomechanical characteristics associated with falls among people with RA.

## Methods

### Design

We conducted a prospective study with nested case–control biomechanical analysis.

### Subjects

A total of 600 men and women with RA attending a rheumatology clinic at the Norfolk and Norwich University Hospital (NNUH) were invited to take part by letter of invitation from July 2012 to January 2014. Participants were eligible if they were ≥60 years of age with a diagnosis of RA, under the care of a rheumatologist and provided written informed consent. Participants were excluded if they presented with severe and enduring mental health problems or other comorbidities that in the clinician’s judgement made them unable to adhere with the protocol.

### Baseline assessment

Participants were asked to complete a questionnaire recording age, gender, relationship status (married/single), employment status (employed/retired/other), visual analogue scale (VAS) pain score, VAS fatigue score, self-reported dizziness experienced, European Quality of Life 5-Dimensions questionnaire (EQ-5D) [20] and previous 12 month history of falls. The Phone-FITT [21] was used to assess physical activity (frequency, intensity, time and type). In addition, participants were asked to give details of their medication use and whether they had been diagnosed with Parkinson’s disease or stroke.

### Ascertainment of falls

Participants returned a postcard monthly for 12 months to report the timing, circumstances and severity of falls. In ascertaining falls, participants were asked to report any slip or trip in which they lost balance and landed on the floor, ground or lower level. This definition of falls follows the recommendations of the Prevention of Falls Network Europe (PROFANE) and the Outcomes Consensus Group documented in Lamb *et al.* [1]. The recommendations include that a fall should be defined as ‘an unexpected event in which the participants come to rest on the ground, floor, or lower level’. This was included in our questionnaire wording.

### Nested sample

A group of 30 ‘fallers’ (*n* = 30) who reported having had one or more falls in the 12 month interval and 30 who reported that they had not fallen were invited to attend a biomechanical assessment. The fallers were selected at random from the base cohort; each faller was matched one to one to a non-faller, stratifying on age and gender.

### Biomechanical assessment

Participants in the nested study were assessed with the following biomechanical measures.

#### Balance

Force plate data were collected at 1000 Hz using Vicon Nexus software (Vicon, Oxford, UK). Data were filtered in Vicon Nexus software using a fourth-order zero-lag Butterworth filter with 5 Hz cut-off. The data were processed using a custom written Matlab script (MathWorks, Natick, MA, USA) to extract values for statistical analysis. Data were split into *x*/*y* components with the mean subtracted to account for position on the force plate. The mean of the three trials, each for 30 s with participants eyes open and then eyes closed, was calculated. The mean (s.d.) for the lateral and forward–backward directions of postural sway was calculated and the root mean square (RMS) for the deviations from the mean position served as the extent of postural sway (COP-RMS). The parameters extracted were anterior–posterior (AP) postural sway (COP-RMS), medio–lateral (ML) postural sway (COP-RMS), AP sway range, ML sway range, resultant velocity and (6) resultant path length.

#### Muscle strength

Participants’ knee flexor and extensor muscle strength was assessed using isokinetic and isometric dynamometry (Cybex NORM 770, Cybex International, New York, NY, USA). During isometric testing the lower limb was placed and secured with straps so that the tested knee was maintained in a fixed position during testing. Knee flexion at 90°, 60° and 30° was tested for both knees. During isokinetic testing, angular velocities were set at three speeds: 120, 90 and 60°/s through the available voluntary range of movement (ROM) for individual participants. Three maximal contractions for each condition were performed with a 15 s rest between each contraction. The participant was asked to push or pull as hard as they could. During the contraction the researcher provided verbal encouragement and the participant was able to see his/her progress as displayed on the screen. A practice contraction for each movement was completed prior to the test contraction. The highest peak torque for each of the six conditions was used for analysis. The parameters extracted were peak torque; limb asymmetry, defined as the difference in peak torque between limbs as a percentage of the most powerful limb; and knee flexion/extension muscle imbalance, calculated as the ratio of the ipsilateral hamstring and the ipsilateral quadriceps concentric peak torque (H:Q ratio). Asymmetry was defined as the difference in peak torque between limbs as a percentage of the most powerful leg [(peak torque of weak limb − peak torque of strong limb)/peak torque of strong limb) × 100. Using this analysis, the value 0% represents equal strength between the lower limbs.

#### Gait

An eight-camera three-dimensional gait motion analysis system (Vicon, Oxford, UK) was used to assess gait. Fourteen reflective markers (25 mm) were bilaterally attached onto the participant’s skin using the Plug-In-Gait model (Vicon) based on the Newington model [22]. All participants were asked to walk at a self-selected speed, wearing flat shoes, along a 7 m walkway. To ensure that three left and three right foot contacts were recorded, it was necessary to ask participants to undertake repeated walks. Two floor-embedded forces plates (Bertec 4060, Bertec, Columbus, OH, USA) were used to collect ground reaction forces. Joint moments were normalized to body weight. Discrete gait kinematic and kinetic parameters were obtained from a mean of three trials for each side. These were ROM and peak extension and flexion at the hip and knee; peak abduction of the initial swing at the hip and peak varus in mid stance at the knee; and ROM, peak plantarflexion in pre-swing/early swing and dorsiflexion at late single support at the ankle.

### Statistical analysis

Cohort characteristics and biomechanical data were assessed using descriptive statistics. To assess the association between falls and clinical characteristics, a multivariate logistic regression analysis was undertaken to determine which variables influenced the occurrence of one or more falls in the previous 12 months prior to the baseline questionnaire. Physical activity was analysed as two composite variables (physical activity home and physical activity recreation). This was achieved by summing the frequency, duration and intensity for each ‘type’ of activity and then summing all the activities to make the two composite variables (physical activity home and physical activity recreation). Stepwise regression (using backwards elimination) was used to remove the non-significant variables from the initial model.

#### Biomechanical study

For each balance measurement and the dynamometry data, an unpaired Student’s *t*-test was performed to assess mean differences between the two groups. Gait data were analysed using a two-way repeated measures analysis of variance (ANOVA) with side (left and right limb) as the within-group factor and group (fallers/non-fallers) as the between-group factor. Intergroup differences with 95% CIs were estimated from the ANOVA model. Differences were considered statistically significant for *P*-values <0.05. All statistical analyses were calculated using Stata 14.2/SE (StataCorp, College Station, TX, USA).

#### Sample size

A target sample size of 600 was chosen so as to detect an odds ratio (OR) with 95% CI associated with a 10% exposure of 1.7, assuming a baseline annual falls rate of 35% and assuming a 50% non-participation rate. In the nested case–control study, the sample size of 30 fallers and 30 non-fallers was determined *a priori* to detect a possible 0.5 s.d. difference in kinematic measures (i.e. stride length, swing:stance ratio, knee ROM) between cases and controls, with a power of 80% and a significance of *P* < 0.05.

## Results

### Participants

Of the 600 people who were invited, 436 people agreed to take part (response rate 73%). The demographic characteristics of the cohort are presented in Table 1. In total, 200 (46%) reported at least one fall in the 12 month period prior to the questionnaire. The mean age of those who reported a fall was 73.2 years (s.d. 7.9) and 71.4 years (s.d. 6.8) for those who did not report a fall. Seventy-three percent of the falls group were female, compared with 65% for the non-falls group. Those in the falls group reported higher pain scores (VAS 48.4 *vs* 34.7) and fatigue (VAS 52.4 *vs* 41.5) and a greater proportion experienced dizziness (76% *vs* 49%). There was no difference between groups in physical activity levels at home (Phone-FITT 26.9 *vs* 29.9) and engagement in recreational activities (Phone-FITT 12.5 *vs* 13.9).

**Table 1:** Baseline characteristics for those who had experienced one or more falls and those who had not experienced a fall in the previous 12 months prior to administration of the questionnaire

Characteristics	Falls group	Non-falls group
*N*	200	236
Age, mean (s.d.), years	73.2 (7.9)	71.4 (6.8)
Gender (female), %	73	65
Employment status, %		
Employed	5	11
Retired	89	86
Other, including caregiver	7	3
Relationship status, %		
Single	34	21
Married	66	79
Physical activity (Phone-FITT data), mean (s.d.) [21]		
Home	26.9 (12.0)	29.9 (12.2)
Recreation	12.5 (11.6)	13.9 (19.2)
Medical status		
Pain VAS, mean (s.d.)	48.4 (25.9)	34.7 (25.2)
Fatigue VAS, mean (s.d.)	52.4 (24.6)	41.5 (24.6)
Dizziness, %	76	49
Four or more medicines each day, %	77	60
Medicine for anxiety/depression, %	34	16
Stroke or Parkinson’s disease, %	6	2

### Clinical factors associated with falls

In the multivariate model, five variables were retained to indicate a difference between the groups in the final model after stepwise elimination (Table 2). Compared with those who did not fall, those who reported one or more falls in the past year were older [OR 1.04 (95% CI 1.01, 1.07), *P* = 0.05], not married [OR 1.73 (95% CI 1.06, 2.86), *P* = 0.03], had higher pain scores [OR 1.02 (95% CI 1.01, 1.03), *P* < 0.01], experienced dizziness [OR 2.46 (95% CI 1.56, 3.91), *P* < 0.01] and were taking psychotropic medications [OR 1.82 (95% CI 1.09, 3.050, *P* = 0.02]. There was no significant relationship between falling and gender, employment status, home-based physical activity, recreational physical activity, VAS fatigue, a diagnosis of Parkinson’s disease, a previous stroke or taking four or more medications (*P* > 0.05).

**Table 2:** Results for the initial and final regression models to assess risk factors for falling in people with RA from the baseline cohort study data

Characteristics	Initial regression model [*n* = 388 (89%)]		Final regression model [*n* = 410 (94%)]	
	OR (95% CI)	*P*-value	OR (95% CI)	*P*-value
Age	1.03 (0.99, 1.06)	0.119	1.03 (1.00, 1.06)	0.051
Gender (male)	Reference			
Gender (female)	1.20 (0.73, 1.97)	0.483		
Relationship (married)	Reference			
Relationship (single)	1.75 (1.03, 2.98)	0.040	1.73 (1.06, 2.86)	0.030
Employment (employed)	Reference			
Employment (retired)	0.99 (0.40, 2.63)	0.987		
Employment (other)	2.25 (0.60, 9.10)	0.240		
Physical activity (home)	0.99 (0.97, 1.01)	0.415		
Physical activity (recreation)	1.00 (0.99, 1.02)	0.605		
Pain VAS	1.02 (1.01, 1.03)	0.004	1.02 (1.01, 1.03)	<0.001
Fatigue VAS	1.00 (0.98, 1.01)	0.441		
Dizziness (yes)	2.32 (1.43, 3.80)	<0.001	2.46 (1.56, 3.91)	<0.001
Four or more medicines each day (yes)	1.21 (0.71, 2.05)	0.491		
Medicine for anxiety/depression (yes)	1.67 (0.95, 2.94)	0.074	1.82 (1.09, 3.05)	0.023
Stroke or Parkinson’s disease (yes)	1.68 (0.52, 6.12)	0.402		

### Biomechanical factors associated with falls

The characteristics of the 30 fallers and 30 non-fallers who took part in the nested case–control gait laboratory study are presented in Table 3. The median number of falls was 2.00 (interquartile range 1.00–3.75). There was no difference in BMI, employment status and relationship status between groups. There was no difference in knee flexion and extension peak torque at 120°, 90° and 60° knee ranges. Differences in biomechanical parameters assessed among those with and without a fall in the previous year are outlined below.

**Table 3:** Characteristics of the longitudinal cohort of subjects who completed biomechanical tests

Characteristics	Falls group	Non-falls group
*N*	30	30
Age, years, mean (s.d.)	72.4 (7.3)	72.5 (7.0)
BMI, mean (s.d.)	28.1 (5.4)	26.2 (4.5)
Body weight, kg, mean (s.d.)	75 (16)	72 (14)
Height, cm, mean (s.d.)	163 (10)	166 (10)
Gender (female), %	50	47
Employment, %		
Employed	0	7
Retired	73	73
Other	27	20
Relationship, %		
Single	3	13
Married	90	87
Divorced/separated/widowed	7	0
DAS [22]		
Swollen joint count, median (IQR)	2.5 (1–4)	1 (0–1)
Tender joint count, median (IQR)	2 (0–8)	1 (0–5)
Patient global health, mean (s.d.)	56.5 (24.9)	72.1 (19.8)
EQ-5D, mean (s.d.) [20]		
Utility	0.65 (0.27)	0.78 (0.20)
VAS	70 (19.25)	76.7 (17.59)
Peak torque, mean (s.d.)		
Maximum flexion 120°	40.6 (13.7)	41.4 (15.7)
Maximum flexion 90°	53.2 (15.0)	52.8 (18.2)
Maximum flexion 60°	64.0 (19.1)	62.9 (22.2)
Maximum extension 120°	109.5 (31.0)	103.8 (34.4)
Maximum extension 90°	97.8 (28.7)	95.9 (31.6)
Maximum extension 60°	74.8 (25.3)	77.1 (29.7)

IQR: interquartile range.

#### Balance

The fallers had a significantly higher postural sway (COP-RMS) and sway range in both the AP (5.2 *vs* 4.1 mm, *P* = 0.03; 28.3 *vs* 21.3 mm, *P* = 0.02) and ML (3.3 *vs* 2.3 mm, *P* = 0.01; 19.7 *vs* 12.7 mm, *P* = 0.02) directions compared with non-fallers during standing with eyes open (Table 4). Both groups had increased sway with eyes closed, but there was no significant difference between the groups for this measure.

**Table 4:** COP balance measures in fallers and non-fallers in the longitudinal cohort during quiet standing with eyes closed and eyes open

Balance measures	Non-fallers (*n* = 29)	Fallers (*n* = 29)	*P*-value
Eyes open			
AP sway (COP-RMS), mm	4.1 (1.9)	5.2 (2.6)	0.03
AP sway range, mm	21.3 (9.1)	28.3 (14.2)	0.02
ML sway (RMS), mm	2.3 (1.3)	3.3 (2.0)	0.01
ML sway range, mm	12.7 (7.6)	19.7 (14.8)	0.02
Resultant velocity, mm/s	5.7 (4.0)	6.1 (68.0)	0.32
Resultant path length, mm	170.7 (119.1)	183.3 (82.4)	0.32
Eyes closed			
AP sway (COP-RMS), mm	6.5 (8.6)	6.3 (5.0	0.46
AP sway range, mm	27.0 (15.5)	31.2 (11.8)	0.12
ML sway (RMS), mm	4.6 (9.9)	4.0 (5.4)	0.39
ML sway range, mm	15.5 (12.4)	19.0 (10.4)	0.13
Resultant velocity, mm/s	8.2 (6.1)	9.0 (4.7)	0.28
Resultant path length, mm	244.7 (183.5)	269.9 (140.8)	0.28

Values are presented as mean (s.d.).

#### Muscle strength

Twenty-three fallers and 28 non-fallers completed the isokinetic tests and 25 fallers and 30 non-fallers completed the isometric tests. Tests were not completed either due to pain in their knee or physical inability. Fallers had a lower isokinetic peak torque at each speed during flexion compared with non-fallers (Table 5). At 60° this is significantly lower (48.23 *vs* 57.95 Nm, *P* = 0.03). Fallers had a higher isokinetic peak torque at each speed during extension than the non-faller, although the level of significance was not reached. Fallers had a lower isometric peak torque than non-fallers during extension and flexion at each position tested, but this was not statistically significant (Table 5).

**Table 5:** Mean peak isokinetic and isometric torque (Nm) for the fallers and non-fallers for three speeds (120, 90 and 60°/s) and three positions (90° 60° and 30°) for extension at the knee (quadriceps) and flexion at the knee (hamstring)

			Mean peak torque (Nm)					Asymmetry (%)			H:Q ratio		
			Faller		Non-Faller		*P*-value	Faller	Non-Faller	*P*-value	Faller	Non-faller	*P*-value
			Mean n = 23	s.d.	Mean n = 28	s.d.		% n = 19	% n = 26				
Isokinetic	120°/s	Flexion	32.2	20.0	38.0	24.9	0.05	31.0	36.0	0.57	0.97	1.31	0.24
		Extension	44.3	24.3	39.0	24.6	0.16	43.1	38.8	0.64			
	90°/s	Flexion	40.4	22.6	48.7	25.8	0.41	31.2	33.8	0.72	0.82	1.07	0.07
		Extension	55.3	24.7	51.9	25.1	0.12	25.5	29.1	0.64			
	60°/sec	Flexion	48.2	22.9	58.0	28.5	0.03	21.7	31.2	0.15	0.82	1.01	0.09
		Extension	65.6	28.0	64.2	29.3	0.26	29.2	26.3	0.71			
Isometric	90°	Flexion	34.5	14.1	36.8	14.9	0.57	19.3	15.5	0.31			
		Extension	85.8	37.2	95.5	31.1	0.31	21.9	13.1	0.05			
	60°	Flexion	47.4	15.9	48.5	17.7	0.80	16.0	13.9	0.55			
		Extension	77.7	32.3	89.0	30.2	0.19	24.1	13.3	0.02			
	30°	Flexion	57.8	18.7	58.3	20.9	0.93	14.4	12.5	0.51			
		Extension	60.5	28.1	70.2	26.9	0.20	17.7	13.2	0.27			

The fallers had a significantly greater asymmetry during isometric extension at 90° (22% *vs* 13%, *P* = 0.05) and 60° (23% *vs* 13%, *P* = 0.02) compared with the non-fallers (Table 5). No significant differences between the H:Q ratio of fallers and non-fallers was seen during isokinetic contractions (Table 5).

#### Gait

The fallers had a significantly shorter stride length (1.05 *vs* 1.18 m, *P* = 0.04), longer double support time (0.39 *vs* 0.32 s, *P* = 0.04) and reduced percentage time in swing (34.2% *vs* 36.8%, *P* = 0.02) than the non-fallers (Table 6). Fallers had significantly smaller knee ROM through the gait cycle compared with non-fallers (50.3° *vs* 58.3°, *P* < 0.01). This smaller knee ROM at the knee in the fallers was accompanied by smaller knee flexion than the non-fallers (51.6° *vs* 56.0°, *P* < 0.01). In all other parameters, no significant differences between fallers and non-fallers were seen (Table 6).

**Table 6:** Kinematic and temporal-spatial parameters for fallers and non-fallers

Parameters	Non-faller (*n* = 29), mean (s.d.)	Faller (*n* = 24), mean (s.d.)	Mean difference (95% CI)	*P*-value
Hip kinematic				
Flexion–extension ROM	38.3 (5.5)	36.1 (7.8)	2.9 (−0.75, 6.53)	n.s.
Hip extension	−11.9 (7.6)	−8.5 (10.5)	−3.3 (−8.23, 1.61)	n.s.
Hip flexion	26.3 (6.8)	27.6 (7.0)	−0.42 (−4.14, 3.3)	n.s.
Peak abduction at initial swing	6.0 (3.9)	5.3 (4.7)	0.08 (−2.23, 2.39)	n.s.
Extension in the loading response	0.52 (0.17)	0.52 (0.16)	−0.03 (−0.13, 0.08)	n.s.
Flexion in late stance	−0.81 (0.37)	−0.75 (0.26)	−0.1 (−0.3, 0.1)	n.s.
Abduction in mid-stance	0.89 (0.20)	0.93 (0.21)	−0.09 (−0.22, 0.03)	n.s.
Adduction at terminal stance	−0.08 (0.07)	−0.07 (0.05)	−0.01 (−0.05, 0.02)	0.24
Knee kinematic				
Flexion–extension ROM	58.3 (5.5)	50.3 (12.7)	9.44 (4.27, 14.61)	<0.001
Knee extension	−2.3 (5.0)	1.3 (7.3)	−2.89 (−6.21, 0.43)	n.s.
Knee flexion	56.0 (5.6)	51.6 (10.6)	6.55 (2.12, 10.95)	0.004
Peak valgus/varus in mid-swing	9.5 (7.4)	9.7 (10.6)	−2.64 (−7.44, 2.16)	n.s.
Peak valgus/varus in stance	2.4 (4.1)	2.6 (7.6)	−0.7 (−3.9, 2.49)	n.s.
Extensor in the loading response	0.48 (0.22)	0.52 (0.21)	−0.01 (−0.14, 0.12)	n.s.
Flexor in terminal stance	−0.33 (0.1)	−0.31 (0.12)	− 0 (−0.06, 0.07)	n.s.
Valgus in mid-stance	0.40 (0.16)	0.44 (0.27)	−0.08 (−0.2, 0.04)	n.s.
Varus in terminal stance/initial swing	−0.07 (0.03)	−0.06 (0.03)	0 (−0.02, 0.02)	n.s.
Ankle kinematic				
Dorsiflexion–plantarflexion ROM	22.8 (5.0)	23.9 (5.7)	−0.95 (−3.81, 1.92)	n.s.
Plantarflexion in pre-swing/early swing	12.7 (5.0)	13.5 (7.0)	−0.6 (−3.87, 2.67)	n.s.
Dorsiflexion late single support	−10.2 (7.3)	−10.4 (9.2)	0.35 (−4.07, 4.77)	n.s.
Plantarflexion at pre-swing	1.24 (0.21)	1.19 (0.25)	0.02 (−0.11, 0.16)	0.330
Dorsiflexion in the loading response	−0.18 (0.1)	−0.18 (0.1)	−0.01 (−0.07, 0.05)	n.s.
Temporal-spatial parameters				
Speed, m/s	1.0 (0.2)	0.9 (0.3)	−0.13 (0.26, 0.00)	0.054
Stride length, m	1.2 (0.2)	1.1 (0.3)	−0.13 (−0.25, −0.01)	0.039
Step length, m	0.6 (0.1)	0.5 (0.1)	−0.06 (−0.12, 0.00)	0.057
Cadence, steps/min	10.3 (11.5)	10.0 (9.5)	10.30 (−5.60, 6.20)	0.266
Single support, s	0.4 (0.1)	0.4 (0.1)	0.00 (−0.06, 0.06)	0.205
Double support, s	0.3 (0.1)	0.4 (0.1)	0.10 (0.05, 0.16)	0.041
Swing, %GC	36.8 (1.9)	34.2 (3.9)	2.61 (0.93, 4.25)	0.019

GC: gait cycle; n.s.: not significant.

## Discussion

Our analysis showed that people with RA who are older and unmarried and who have higher pain scores and dizziness and who take psychotropic medications are at greater risk of falls. While physical activity performance was not associated with the risk of falls, those who fell appeared to have a characteristic biomechanical signature with increased postural sway, reduced peak torque and strength and gait differences showing a shorter stride length, reduced swing phase and reduced knee ROM through the gait cycle.

The findings in our cohort of people with RA are supported by a number of other cross-sectional [2, 23] and longitudinal studies in cohorts of similar ages [24]. Being single was also reported as a significant factor for the occurrence of falls. This too has been previously reported in the English population [25]. However, due to the data collection processes, we were unable to explore whether this finding reflects previous marital histories (such as long-term first marriages, never married, widowed, divorced, long-term partners), which have been reported as important distinctions within health and mortality outcomes in older people [26].

While previous studies of the biomechanics of falling in RA have been limited in their scope of assessment, taken together, their findings are in broad agreement with our own. Hayashibara *et al.* [27] reported that postural sway was greater for people with RA who had fallen compared with non-fallers. Rome *et al*. [28] reported that people with RA have poorer dynamic and static postural control than age-matched non-RA individuals. Our RA fallers and non-fallers showed similar results to Rome *et al.*’s study, although we report slightly poorer results for AP and ML sway range during eyes open. This may be due to differences in methodology. Rome *et al*. [28] presented the participants with a target to focus on during eyes open. This was not part of the protocol in our study. Our findings indicate that non-fallers have better use of their visual systems.

Both groups show a strength imbalance across quadriceps and hamstrings. Both fallers and non-fallers had a significantly higher H:Q ratio in their weaker leg compared with their stronger leg during isokinetic contractions. The increase in the H:Q ratio in the fallers and non-fallers in their weaker leg was due to a lower peak torque in the quadriceps. Given these issues around imbalance, general lower limb strength training intervention through physical activity programmes may not necessarily be the most appropriate exercise intervention for this population. Targeting such imbalance through specific muscle-based exercise programmes has a plausible physiological rationale.

This study is the first to investigate the risk of falls and physical activity participation in people with RA. The Phone-FITT tool was used due to its reported reliability and validity [21]. This self-reported tool provides valuable data on both home-based physical activity and recreational physical activity performance. This is important for this population based on their potentially wide-ranging levels of physical activity pursuits [29]. The data indicated that neither home-based nor recreational physical activity were significantly associated with the risk of falls. While previous studies have suggested that strength and balance are associated with a reduced risk of falls in people with osteoarthritis [30], it appears that this may not relate directly to physical participation, which assesses multiple components of physical function. Given the identified biomechanical factors that are associated with falls, targeting biomechanical deficits rather than simply promoting more global physical activity engagement would be indicated from these findings.

A notable strength of this study is the recruitment of a representative RA cohort as the basis for the nested study. Previous studies of biomechanical factors involved in falls have been based on small selective samples, with falls history based on recall. Our sample was selected from a large group of clinic attenders with RA in whom falls were identified prospectively. Limitations of the study include the fact that not all participants completed the dynamometry tests due to having a painful knee or were unable to create any level of torque due to weakness. Participants were recruited from a regional rheumatology service; accordingly, they may have presented with more severe disease activity compared with those from the community. The relatively small number of cases in the biomechanical evaluation cohort precluded any analysis of the relationship between specific clinical or biomechanical features and the frequency of falling because of insufficient power. We were also unable to assess disease activity using the DAS [31] at baseline or during follow-up in the cohort study, given the self-completed nature of the questionnaire. We note that pain was associated with the risk of falls in the survey study, providing an indication that disease severity may be related to falls. However, given the small sample, this finding should be considered exploratory.

## Conclusions

Characteristic clinical and biomechanical factors have been identified as being associated with falls in people with RA. Our findings suggest that physical activity performance alone may be insufficient to reduce falls and that targeting interventions to address specific biomechanical deficits for those individuals with RA at increased risk for falls would be appropriate.
