# Correction to: Clinical and biomechanical factors associated with falls and rheumatoid arthritis: baseline cohort with longitudinal nested case–control study

**DOI:** 10.1093/rheumatology/kead043

**Published:** 2023-02-03

This is a correction to: Toby O Smith, Celia Clarke, Jacob Wells, Jack R Dainty, Laura Watts, Max Yates, Valerie M Pomeroy, Emma Stanmore, Terence W O’Neill, Alexander J Macgregor, Clinical and biomechanical factors associated with falls and rheumatoid arthritis: baseline cohort with longitudinal nested case–control study, *Rheumatology*, Volume 61, Issue 2, February 2022, Pages 679–687, https://doi.org/10.1093/rheumatology/keab388

The author list has been updated to add Jacob Wells as third author.

